# Population pharmacokinetics of quinine in pregnant women with uncomplicated *Plasmodium falciparum* malaria in Uganda

**DOI:** 10.1093/jac/dku228

**Published:** 2014-06-25

**Authors:** Frank Kloprogge, Vincent Jullien, Patrice Piola, Mehul Dhorda, Sulaiman Muwanga, François Nosten, Nicholas P. J. Day, Nicholas J. White, Philippe J. Guerin, Joel Tarning

**Affiliations:** 1 Centre for Tropical Medicine, Nuffield Department of Clinical Medicine, University of Oxford, Oxford, UK; 2 Mahidol-Oxford Tropical Medicine Research Unit, Faculty of Tropical Medicine, Mahidol University, Bangkok, Thailand; 3 Université Paris Descartes, INSERM U663, Assistance Publique-Hôpitaux de Paris, Hôpital Saint-Vincent de Paul, Paris, France; 4 Epicentre, Paris, France; 5 Mbarara University of Science & Technology, Mbarara, Uganda; 6 Epicentre, Mbarara, Uganda; 7 Malaria Group, Center for Vaccine Development, University of Maryland School of Medicine, Baltimore, MD, USA; 8 Shoklo Malaria Research Unit, Faculty of Tropical Medicine, Mahidol University, Mae Sot, Thailand

**Keywords:** population models, *P. falciparum*, NONMEM

## Abstract

**Objectives:**

Oral quinine is used for the treatment of uncomplicated malaria during pregnancy, but few pharmacokinetic data are available for this population. Previous studies have reported a substantial effect of malaria on the pharmacokinetics of quinine resulting from increased α-1-acid glycoprotein levels and decreased cytochrome P450 3A4 activity. The aim of this study was to investigate the pharmacokinetic properties of oral quinine in pregnant women with uncomplicated malaria in Uganda using a population approach.

**Methods:**

Data from 22 women in the second and third trimesters of pregnancy with uncomplicated *Plasmodium falciparum* malaria were analysed. Patients received quinine sulphate (10 mg of salt/kg) three times daily (0, 8 and 16 h) for 7 days. Plasma samples were collected daily and at frequent intervals after the first and last doses. A population pharmacokinetic model for quinine was developed accounting for different disposition, absorption, error and covariate models.

**Results:**

Parasitaemia, as a time-varying covariate affecting relative bioavailability, and body temperature on admission as a covariate on elimination clearance, explained the higher exposure to quinine during acute malaria compared with the convalescent phase. Neither the estimated gestational age nor the trimester influenced the pharmacokinetic properties of quinine significantly.

**Conclusions:**

A population model was developed that adequately characterized quinine pharmacokinetics in pregnant Ugandan women with acute malaria. Quinine exposure was lower than previously reported in patients who were not pregnant. The measurement of free quinine concentration will be necessary to determine the therapeutic relevance of these observations.

## Introduction

Malaria caused ∼660 000 deaths in 2010. Pregnant women are especially vulnerable to malaria, with increased morbidity and mortality depending on the intensity of transmission, and with intrauterine growth retardation at all levels of malaria transmission.^1^ Artemisinin-based combination therapies and parenteral artesunate are now recommended by the WHO as first-line treatments for uncomplicated and severe *Plasmodium falciparum* malaria, respectively, while quinine is recommended during the first trimester of pregnancy.^2^ With the continued limited availability of artemisinin-based therapies, which are more effective and better tolerated, quinine is still widely used and remains part of the national guidelines in many countries as a second-line treatment for uncomplicated malaria, a first-line treatment for severe malaria and a treatment for malaria during pregnancy.

During acute malaria, quinine plasma concentrations are substantially higher than in the convalescence phase of the treatment.^3–5^ The reduced metabolic clearance of quinine in acute illness results from decreased cytochrome P450 (CYP) 3A4 activity.^6^ Increased α-1-acid glycoprotein concentrations in the acute phases of the disease result in an increased plasma protein binding of quinine, which contributes to the decreased volume of distribution and the increased quinine concentration during the acute phase of the disease.^7,8^

The pharmacokinetic properties of drugs may be altered during pregnancy as a result of physiological alterations (including reduced gut motility and drug metabolism) and changes in body composition.^9–11^ A comparison using a non-compartmental analysis of quinine pharmacokinetics in pregnant (*n* = 8) and non-pregnant (*n* = 8) women with uncomplicated *P. falciparum* malaria in Sudan did not show a significantly different exposure or elimination *t*_½_ after intravenous administration.^12^ However, a study in pregnant Thai women with severe *P. falciparum* malaria (*n* = 10) reported a shorter elimination *t*_½_ (11.3 versus 16.0 and 18.2 h) and a smaller apparent volume of distribution (0.96 versus 1.67 and 1.18 L/kg) compared with previously studied non-pregnant patients with malaria (i.e. patients with uncomplicated *P. falciparum* malaria and cerebral malaria) after intravenous administration of quinine.^13,14^ This suggested that pregnancy might affect the distribution of intravenous quinine and therefore the terminal elimination *t*_½_ of, but not the total exposure to, the drug.

The aim of this study was to evaluate the population pharmacokinetics of orally administered quinine in women in the second and third trimesters of pregnancy with uncomplicated *P. falciparum* malaria in Uganda.

## Methods

### Study design

This pharmacokinetic study was conducted in the Mbarara National Referral Hospital (MNRH) antenatal clinic in Uganda and nested into a larger efficacy study published elsewhere.^15^ A non-compartmental analysis of quinine plasma concentration–time data after the first dose has previously been published.^16^ The trial was registered at ClinicalTrials.gov (NCT00495508) and patients in the quinine pharmacokinetic study arm were recruited from 19 February to 23 July 2008. Ethical approval was obtained from the Mbarara University Faculty of Medicine Research and Ethics Committee, the Mbarara University Institutional Ethics Committee, the Uganda National Council for Science and Technology (ethics committee) and the ‘Comités de Protection des Personnes’ (Ile de France XI, France).

Inclusion criteria were an estimated gestational age (EGA) of at least 13 weeks (confirmed by ultrasound or fundal height and the Dubowitz score at delivery if ultrasound was not available), residence in the Mbarara Municipality (a radius of 15 km from MNRH) and *P. falciparum* mixed or mono-infection (detected by microscopy). The exclusion criteria were a known allergy to artemisinin derivatives, lumefantrine or quinine, an inability to comply with the specified follow-up schedule, severe anaemia (haemoglobin <7 g/dL), signs or symptoms of severe malaria requiring parenteral treatment or *P. falciparum* parasitaemia above 250 000 parasitized red cells/μL. Patients were enrolled if written informed consent was obtained and if they fulfilled all the inclusion criteria and none of the exclusion criteria.

Oral quinine sulphate (Remedica, Limassol, Cyprus; 300 mg of salt per tablet; 10 mg of salt/kg per dose) was administered under supervision three times daily (0, 8 and 16 h) for 7 days. A full or a half replacement dose was given if the dose was vomited within 30 min or between 30 and 60 min, respectively. If the dose was vomited again within 30 min, the patient was withdrawn from the study and treated with rescue treatment [artemether/lumefantrine (Coartem^®^), four tablets twice daily for 3 days]. Venous blood samples (2 mL) were collected in heparinized tubes at 0, 1, 2, 3, 4, 8, 16, 24, 48, 72, 96, 120, 144, 160, 161, 162, 163, 164, 168, 170, 172, 176 and 184 h after the first dose. Blood samples were centrifuged for 5 min at 1400 **g** and plasma was stored at −70°C or in liquid nitrogen until analysis.

### Quinine concentration measurements

Quinine drug analysis was performed using liquid chromatography with fluorimetric detection. A volume of 50 μL of sodium hydroxide 0.1 M and 50 μL of the internal standard (7.5 μg/L hydroquinidine) were added to 50 μL of plasma. Liquid/liquid extraction was performed with 4 mL of dichloromethane:isopropylic alcohol (80 : 20). After 10 min of mixing, the samples were centrifuged and the supernatant was separated and evaporated under a stream of nitrogen. The dry residue was reconstituted with 100 μL of the mobile phase and 30 μL was injected into the chromatographic system. Chromatographic separation was performed on a Cluzeau C8+ satisfaction column (250 × 3 mm; 3 μm; Sainte-Foy la Grande, France) with a mobile phase consisting of dihydrogen potassium phosphate 0.1 M:acetonitrile:acetic acid (695 : 300 : 5). The retention times of quinine and the internal standard (Roussel Uclaf, Paris, France) were 4.9 min and 6.1 min, respectively. The excitation and emission wavelengths were 350 and 440 nm, respectively. The recovery was between 76% and 80% within the calibration range of 1–10 μg/mL. Duplicates of quality control samples were analysed at three concentrations: 2, 6 and 8 μg/mL. Overall accuracy (bias) and precision (relative standard deviation, RSD) were less than 5.0% and 9.9%, respectively, and the lower limit of quantification was set to 1 μg/mL.

### Pharmacokinetic analysis

NONMEM v.7.2 (ICON Development Solutions, Ellicott City, MD, USA) with a G-Fortran compiler (Free Software Foundation, Boston, MA, USA) on a Windows 7 operating system (Microsoft Corporation, Seattle, WA, USA) was used for modelling and simulation. The first-order conditional estimation method with interaction and subroutine ADVAN5 TRANS1 was used during model-building.^17^ Post-processing and automation was performed using Perl-speaks-NONMEM v. 3.5.3,^18,19^ Xpose v. 4^20^ and R v. 2.15.1 (The R Foundation for Statistical Computing, Vienna, Austria).

The objective function value (OFV) (computed as minus twice the log likelihood of the data), physiological plausibility and goodness-of-fit (GOF) diagnostics were used to evaluate competing models. A fall in OFV (ΔOFV) of 3.84 or more was considered a significant (*P* = 0.05) improvement in the fit of the model after the introduction of one new parameter (one degree of freedom) into a hierarchical model.

Plasma quinine concentrations were modelled in their natural logarithms and quinine sulphate doses (molecular weight of 782.96 g/mol) were converted into the quinine base equivalent (molecular weight of 324.42 g/mol). Several combinations of absorption models (first-order, first-order with lag-time and transit absorption), distribution models (one-, two- and three-compartment distribution), variability models [inter-individual variability (IIV) and inter-occasion variability (IOV)] and residual variability models (additive, proportional and a combination of the two) were assessed.

The best-performing structural base model was used for covariate model-building. The influence of a disease effect was assessed based on prior information.^3–5,7,8^ Daily parasite counts were evaluated in terms of a time-varying covariate effect on the absorption rate constant, elimination clearance and relative bioavailability, respectively. Three different implementations of this disease model were tried (i.e. the last observed parasite count carried forward, the linear interpolation of the observed parasite counts and a disease model for parasitaemia). Each version of the disease model was evaluated with a linear, exponential and power covariate–parameter relationship. Body weight was subsequently evaluated as an allometric function on all clearance (power coefficients of ¾ and ⅔) and volume (power coefficient of 1) parameters.

All remaining baseline covariates were evaluated formally if the parameter–covariate relationship resulted in a significant correlation (*P* < 0.05 using the Pearson, Spearman or Kendall test, with the ranges not crossing 0) and/or if the relationship was physiologically plausible. The stepwise covariate model-building was conducted using the selected covariates in a forward addition (*P* < 0.05) and backward elimination (*P* < 0.001) approach.^18,21^ A strict cut-off (*P* < 0.001) was used in the backward elimination step due to the small study size (*n* = 22). A linear, exponential and power covariate–parameter relationship was assessed sequentially for continuous covariates, and binary covariates were evaluated as a relative difference between groups. Absorption and disposition models were reconsidered using the final covariate model.

The EGA and trimester of pregnancy were also evaluated separately by a full-covariate model approach. The week of gestation or trimester was implemented simultaneously for all parameters except for relative bioavailability due to identifiability issues when adding a simultaneous covariate effect on clearance, volume and bioavailability. The distribution of the estimated covariate effects for gestational age and trimester were obtained from 200 individual bootstrap runs (stratified by trimester of pregnancy) and visualized using a box and whisker plot (GraphPad Prism v. 6.00; GraphPad Software).

Eta and epsilon shrinkages were calculated to assess the reliability of the individual parameter estimates and the GOF diagnostics.^22^ A stratified (trimester) bootstrap (*n* = 1000) was performed to calculate the non-parametric CIs and relative standard errors of the parameter estimates. The predictive power of the model was examined using visual and numerical predictive checks, i.e. 2000 simulations of each individual plasma sample.^23^ The 95% CIs of the simulated 5th, 50th and 95th percentiles were overlaid with the 5th, 50th and 95th percentiles of the observed data for a visual predictive check.

Monte Carlo simulations (*n* = 1000) were used to evaluate and visualize the effect of disease covariates (i.e. parasitaemia and body temperature on admission) in a typical patient with a body weight of 56 kg receiving 560 mg quinine sulphate as a single dose. Different total parasite biomasses (10^7^, 10^8^, 10^9^, 10^10^ and 10^11^ infected erythrocytes) on admission (a body temperature of 37.1°C) were evaluated, as well as varying degrees of fever (i.e. 36–39°C) on admission (for a total parasite biomass of 1.21^10^ infected erythrocytes). Total exposures to quinine were plotted using GraphPad Prism.

## Results

Twenty-three women in the second and third trimesters of pregnancy were enrolled in this pharmacokinetic study (Table 1). Non-compartmental analysis results of plasma concentration–time data for quinine after the first dose have been published in full elsewhere.^16^ One patient was excluded from the population pharmacokinetic analysis because of an unexplainable mismatch between the dosing history and the plasma quinine concentration–time profile. The treatment was efficacious without any cases of vomiting or reappearance of malaria during the follow-up until delivery or day 42 if this was later. The large efficacy study, which this pharmacokinetic study was part of, showed 1.5%, 1.5%, 2.2% and 4.4% of spontaneous abortions (<20 weeks), intrauterine fetal deaths (>20 weeks), stillbirths and early neonatal deaths (before Week 1 after birth), respectively.^15^ Furthermore, 4.7% and 13.4% of the patients gave birth to babies with a term low birth weight (<2500 g, gestational age at birth ≥37 weeks) or low birth weight (<2500 g, without further specifications).^15^ Four patients were treated with ferrous sulphate and folic acid (*n* = 1), other unknown medicines (*n* = 2) or amoxicillin (*n* = 1) during the course of the quinine treatment and none of these co-medications was expected to affect the pharmacokinetics of the quninine.

**Table 1.** Admission demographics of patients included in the pharmacokinetic study

No. of pregnant women	22
Age (years)	21.0 (18.0–37.0)
Body weight (kg)	56.5 (44.0–71.0)
Gestational age (weeks)	26.0 (13.0–37.0)
No. of patients in the second trimester	12/22 (54.5%)
No. of patients in the third trimester	10/22 (45.5%)
Parity	1 (0–6)
Body temperature (°C)	37.2 (36.0–38.9)
*P. falciparum* (parasites/μL)	2240 (39.0–44,500)
Platelets (10^9^/L)	131 (15.0–313)
Bilirubin (mg/dL)	1.31 (0.310–3.36)
Haematocrit (%)	31.3 (22.1–39.8)
Diastolic blood pressure (mmHg)	63.0 (45.0–80.0)
Haemoglobin (g/dL)	10.4 (7.40–12.7)
Red blood cells (10^12^/L)	3.43 (2.37–4.50)
Neutrophils (10^9^/L)	2.56 (0.550–6.53)^a^
Eosinophils (10^9^/L)	0.0800 (0.0100–0.300)^a^
Basophils (10^9^/L)	0.0300 (0.0100–0.0800)
Lymphocytes (10^9^/L)	2.22 (0.690–3.61)
Monocytes (10^9^/L)	0.645 (0.170–1.34)
Alanine aminotransferase (IU/L)	16.5 (8.00–26.0)
Creatinine (mg/dL)	0.510 (0.380–1.29)

Values are reported as median (range) unless otherwise specified.

^a^Based on 21 patients.

A first-order absorption model followed by a two-compartment disposition model with an additive residual error model on log-transformed data accurately described the quinine data. Simpler structural models resulted in a model mis-specification and more complex models did not result in a significant improvement (*P* > 0.05). Adding IIV (ΔOFV = −34.7) and IOV between doses (ΔOFV = −60.4) to the fixed (100%) relative bioavailability parameter significantly improved the fit of the model.

Parasitaemia, implemented as a time-varying covariate (last observation carried forward), had a significant effect on relative bioavailability (ΔOFV = −88.6) resulting in a 38.9% increase in relative bioavailability per log_10_ parasitaemia. Body weight, allometrically scaled on clearance (a power exponent of ⅔) and volume (a power exponent of 1) parameters, resulted in an improved model (ΔOFV = −4.01).

The following covariate–parameter relationships were identified (physiological plausibility and/or graphical screening) and formally tried in a stepwise covariate approach: body temperature, baseline parasitaemia, age, trimester and EGA on all parameters; alanine aminotransferase on elimination clearance and inter-compartmental clearance; systolic blood pressure on intercompartmental clearance; and body weight on absorption rate constant. The effect of initial body temperature on elimination clearance (an exponential relationship), EGA on bioavailability (a linear relationship) and body weight on absorption rate constant (a power relationship) were significant covariates in the forward addition. However, only an effect of initial body temperature on elimination clearance was retained in the model in the backward elimination (a 51.8% lower elimination clearance at 39°C compared with 36°C).

**Figure 1.**
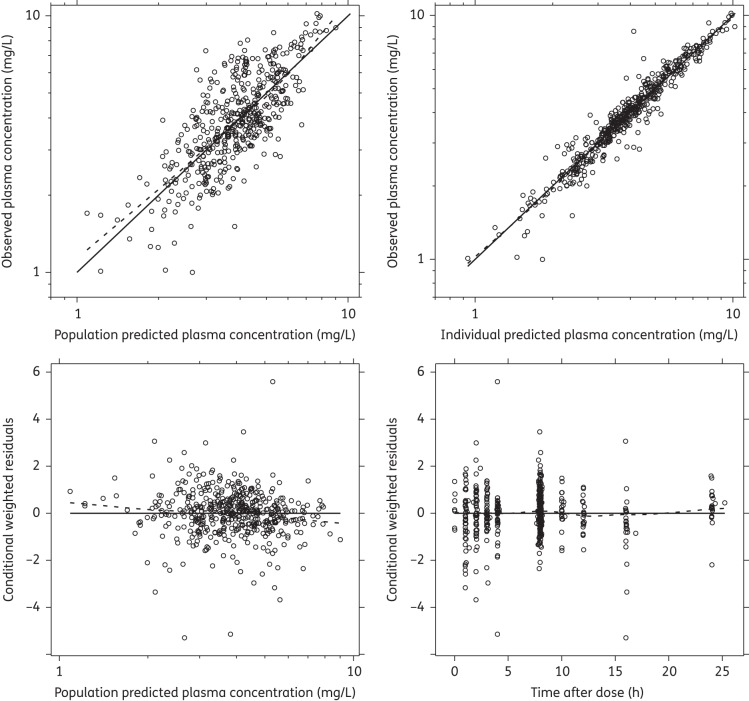
Quinine GOF diagnostics. The continuous black line represents the line of identity, and a local polynomial regression is represented by the broken black line. The observed data are represented by black circles.

**Figure 2.**
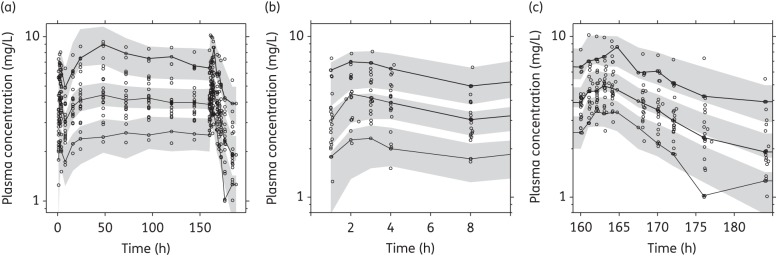
Visual predictive check of the final quinine model. Open circles represent the observed data, continuous lines represent the 5th, 50th and 95th percentiles of the observed data and shaded areas represent the 95% CIs of the simulated 5th, 50th and 95th percentiles. (a) Visual predictive check of all the data. (b) Visual predictive check of the first dose. (c) Visual predictive check of the last dose.

GOF plots from the final model did not show any model mis-specification (Figure 1) and the visual predictive check demonstrated a reasonable predictive power of the model (Figure 2). The numerical predictive check computed 5.45% (95% CI 1.26%–10.5%) and 3.56% (95% CI 1.26%–10.5%) of the observed quinine concentrations above and below the 90% prediction interval, respectively. Eta shrinkages for absorption rate constant, elimination clearance, central apparent volume of distribution, peripheral apparent volume of distribution, bioavailability (IIV) and bioavailability (IOV) were 14.0%, 27.1%, 39.4%, 33.6%, 30.2% and 25.5%–84.5%, respectively. Epsilon shrinkage was 20.1%. High relative standard errors for parameter estimates of intercompartmental clearance (44.6%) and apparent peripheral distribution volume (29.1%) were observed (Table 2).

**Table 2.** Estimates of population pharmacokinetic parameters from the final two-compartment quinine model in pregnant women with uncomplicated *P. falciparum* malaria

Parameter	Population estimate^a^ (% RSE)^b^	95% CI^b^	IIV/IOV (% CV)^a^ (% RSE)^b^	95% CI^b^
k_a_ (1/h)	0.817 (18.8)	0.479–1.03	58.7 (32.7)	40.5–107
CL/F (L/h)	10.4 (4.36)	9.51–11.4	7.69 (65.4)	1.16–47.4
Vc/F (L)	174 (14.0)	112–195	—	—
F (%)	100 (fixed)	—	12.3 (77.0)/21.4 (48.8)	0.170–48.8/19.3–93.3
Q/F (L/h)	10.7 (44.6)	7.06–36.9	—	—
Vp/F (L)	54.3 (29.1)	33.6–112	70.8 (65.3)	8.00–128
Parasitaemia (log_10_) on F (%)	38.9 (9.33)	32.4–47.2	—	—
Temperature on CL/F	−0.243 (21.1)	−0.427 to −0.180	—	—
Additive residual error	0.0158 (41.6)	0.0129–0.156	—	—
Post hoc estimates^c^	All patients	Second trimester	Third trimester	
AUC_0-8_ (mg·h/L)	26.6 (16.0–53.2)	26.2 (16.3–43.8)	27.7 (16.0–53.2)	
AUC_0-12_ (mg·h/L)	36.9 (24.0–49.7)	36.7 (26.8–49.7)	36.9 (24.0–45.3)	
*C*_max_ (mg/L)	4.06 (2.40–7.92)	3.95 (2.54–6.62)	4.16 (2.40–7.92)	
*T*_max_ (h)	3.01 (1.85–8.00)	3.01 (2.00–8.00)	2.75 (1.85–4.34)	
*t*_½_ (h)	15.3 (10.4–30.8)	14.6 (11.0–29.3)	16.1 (10.4–30.8)	
CL/F (L /h/kg)	0.188 (0.113–0.247)	0.196 (0.149–0.238)	0.181 (0.113–0.247)	
V_SS_/F (L/kg)	4.05 (3.53–5.68)	4.07 (3.53–5.68)	4.02 (3.62–5.08)	

k_a_, absorption rate constant; CL/F, elimination clearance; Vc/F, apparent volume of distribution of the central compartment; F, relative bioavailability; Q/F, inter-compartmental clearance; Vp/F, apparent volume of distribution of the peripheral compartment; AUC, total area under the plasma concentration–time curve; *C*_max_, maximum concentration after the last dose, *T*_max_, time after dose to maximum concentration; *t*_½_, elimination *t*_½_; V_SS_, sum of the post hoc apparent central and peripheral volume estimates.

The additive error (σ) variance will essentially be exponential on normal scale data.

^a^Population mean values, IIV and IOV estimated by NONMEM. IIV and IOV are presented as $\text{100}\;\times \;\sqrt{{\text{e}}^{\mathrm{estimated}\;\mathrm{variance}}-\text{1}}$.

^b^The relative standard error (RSE) is calculated as 100 × (standard deviation/mean value) from 1000 runs of a non-parametric bootstrap. The 95% CI is displayed as the 2.5th to 97.5th percentiles of the bootstrap estimates.

^c^Post hoc estimates were calculated as the medians and ranges of the empirical Bayes estimates.

**Figure 3.**
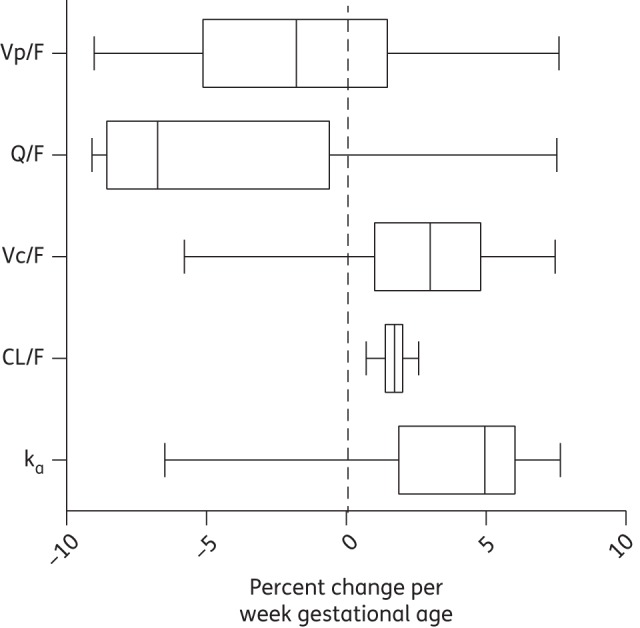
Box and whisker plot visualizing the effect of EGA on pharmacokinetic parameters from 200 bootstraps of the full covariate approach (the boxes represent 25%–75% and the whiskers represent 2.5%–97.5%). Vp/F, apparent volume of distribution of the peripheral compartment; Q/F, inter-compartmental clearance; Vc/F, apparent volume of distribution of the central compartment; CL/F, elimination clearance; k_a_, absorption rate constant.

The bootstrap diagnostics of the full covariate approach (Figure 3) showed a substantial effect of EGA on elimination clearance (a 1.73% median change per week age of gestation) but no significant effects on other parameters. This would result in a 41.5% higher clearance for a woman in Week 37 of her pregnancy compared with a woman in Week 13 of pregnancy. Similar trends were observed in the parameter distributions calculated for women in their second and third trimesters (data not shown).

**Figure 4.**
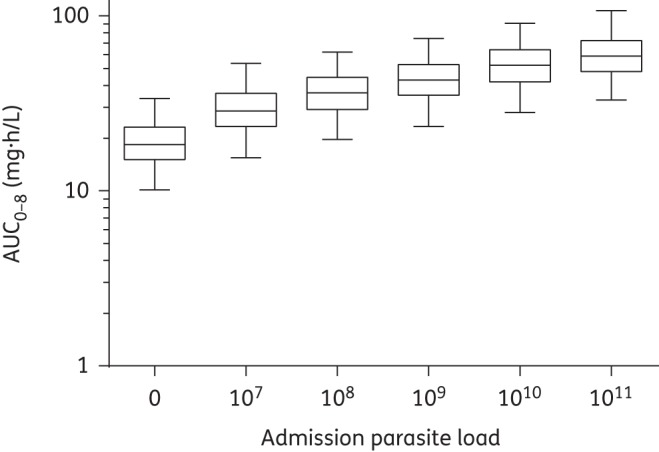
Simulated (*n* = 1000) first-dose exposure (AUC_0-8_) after the administration of 560 mg of quinine sulphate to a typical patient (weighing 56 kg with a body temperature of 37.1°C) at a total parasite load of 0, 10^7^, 10^8^, 10^9^, 10^10^ and 10^11^ infected erythrocytes. Data are represented by box and whisker plots (the boxes represent 25%–75% and the whiskers represent 2.5%–97.5%).

**Figure 5.**
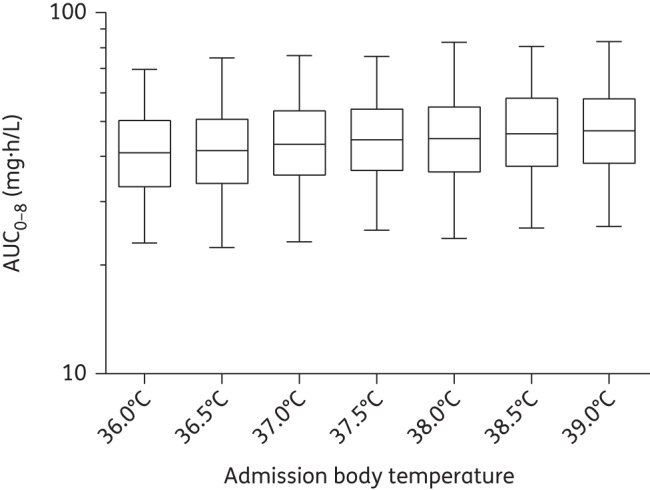
Simulated (*n* = 1000) first-dose exposure (AUC_0-8_) after the administration of 560 mg of quinine sulphate to typical patients (weighing 56 kg and with a total parasite biomass of 1.21^10^ infected erythrocytes) with an admission body temperatures ranging between 36°C and 39°C. Data are represented by box and whiskers plots (the boxes represent 25%–75% and the whiskers represent 2.5%–97.5%).

Simulations showed substantially higher exposures during the acute phase of the disease in patients with a high body temperature on admission and a high total parasite biomass load compared with patients with a lower admission body temperature and total parasite biomass (Figures 4 and 5). The simulated median exposures to quinine during the first 8 h of treatment were 29.2, 36.5, 43.7, 51.1 and 57.6 mg · h/L for patients with a total parasite biomass of 10^7^, 10^8^, 10^9^, 10^10^ and 10^11^ infected erythrocytes, respectively. Exposure to quinine during the first 8 h of treatment also increased with body temperature on admission (Figure 5) at an average of 2.05 mg·h/L per degree Celsius increase between 36°C and 39.0°C.

## Discussion

Quinine is still an important antimalarial drug, but the therapeutic window for the unbound drug is relatively narrow. Minor adverse effects such as tinnitus, dysphoria and nausea (cinchonism) are common and hypoglycaemia is a particular problem in later pregnancy.^24–26^ Despite its intensive use, only limited information on the pharmacokinetics of quinine in pregnant women is available.^12,13^ In this study conducted in pregnant Ugandan women, oral quinine data were analysed using a population pharmacokinetic approach. A limitation of the current study was its relatively small sample size (*n* = 22). However, dense data were collected for all women, which produced a good description of the concentration–time profiles in the women studied. A drawback of small sample sizes is the lack of power to detect true pharmacokinetic differences between groups and they also might not estimate the variability between patients accurately. Due to the small sample size, a parsimonious *P* value of 0.001 was used in the formal backward elimination step of the covariate building in order to avoid false positives. Pharmacokinetic sample collection over the entire duration of treatment offered an advantage to evaluate the disease effect over the treatment course.

The best-performing disposition model consisted of two distribution compartments. Both one^27,28^ and two^29^ disposition models have previously been used to describe the oral and intramuscular pharmacokinetics of quinine. Differences between published disposition models might be caused by different sampling schedules. A first-order absorption model best described the absorption of quinine and more complex absorption models (i.e. first-order with lag-time and transit absorption) did not improve the fit of the model due to a lack of data in the absorption phase.

Body weight was implemented as a continuous covariate on clearance and apparent volume parameters using allometry, which has been shown in previous studies modelling antimalarial drugs.^28,30,31^ A power coefficient of ⅔ on clearance parameters produced a better fit of the model compared with a coefficient of ¾, which is in good agreement with the observed physiology since clearance does not normally scale linearly with body weight.^32^

Malaria affects the pharmacokinetic properties of quinine, resulting in higher total exposures during the acute phase of the disease in proportion to disease severity, but this has not previously been implemented in a population pharmacokinetic quinine model.^3–5,33^ The increase in total quinine peak levels and total quinine exposure with increasing disease severity results from the contracted total apparent volume of distribution (contributed to by the increased α-1-acid-glycoprotein concentrations) and the decreased metabolic clearance (contributed to by reduced CYP 3A4 activity and possible altered α-1-acid-glycoprotein concentrations).^6,7^ A time-varying covariate relationship between parasitaemia and bioavailability was used to describe part of the disease effect in the current study. Parasitaemia was a significant covariate of bioavailability and resulted in a 50.7% higher drug exposure in a typical patient with a total parasite biomass of 10^11^ infected erythrocytes compared with 10^7^ infected erythrocytes. As a consequence of the time-varying aspect, exposure was only affected during the acute phase when parasitaemia was above the limit of detection. Parasite slides were only taken once daily but the exact time was not reported. The last observed parasite count was therefore carried forward and implemented as having a direct effect on the bioavailability of quinine. More complex models (i.e. the interpolation of the parasite counts or a parasite disease model) were evaluated during the model-building process but did not contribute to an improvement in the predictive power of the model based on the current data. It is possible that more frequent parasite counts and more accurate sampling times could have enabled a more mechanistic disease model.

A static covariate relationship between the body temperature on admission and the elimination clearance described the other part of the disease effect in the current study. Body temperature on admission was a significant covariate on elimination clearance and resulted in an ∼15% higher quinine exposure during the first 8 h of treatment in patients with an admission body temperature of 39°C compared with 36°C. A time restriction to the first 24 or 48 h for the covariate–parameter relationship between body temperature on admission and elimination clearance resulted in a significantly worse model (ΔOFV = 16.1 and ΔOFV = 18.7, respectively). This indicates that disease severity, as reflected by body temperature on admission, during the acute phase was still influencing the pharmacokinetics of quinine throughout the entire 7 day treatment.

EGA was a significant covariate on the bioavailability of quinine in the forward addition of covariates (*P* < 0.05) but this covariate could not be retained in the backward step (*P* < 0.001). However, increasing EGA and trimester resulted in a substantial increase in elimination clearance in the full covariate approach (Figure 4). This would result in decreased quinine exposures with increased EGA. Quinine is extensively metabolized by CYP3A4 enzymes and both hepatic and intestinal CYP3A4 activities have been reported to be induced during pregnancy compared with post-partum women.^34–37^ However, no difference in CYP3A4 activity has been reported between the second and third trimesters of pregnancy, which would explain the lack of a covariate effect in this study.^36^ In this study estimated median (range) quinine elimination clearance [0.188 (0.113–0.247) L/h/kg] was higher than previously reported in non-pregnant patients (acute malaria, 0.0906 L/h/kg; convalescent malaria, 0.1602 L/h/kg) and thus quinine exposures were lower.^5^ Lowered total quinine exposures during pregnancy may result in decreased therapeutic efficacy if protein binding is unaffected. However, if these pregnant women had lower protein binding associated with lower acute-phase protein levels as a result of being less ill than in previous studies of uncomplicated malaria in lower-transmission settings, the therapeutic responses might be unaffected. Free quinine concentration measurements are therefore necessary to determine the therapeutic relevance of these of alterations in quinine pharmacokinetics.

The final model was validated using a variety of diagnostic tools (GOF plots, visual predictive checks, numerical predictive checks, bootstrap statistics and shrinkage calculations). Simulation-based predictive checks of the final model resulted in a high predictive power with numerical values close to the theoretical values (i.e. 10% of observations outside the 90% prediction interval).The CIs of the simulated 5th, 50th and 95th percentiles were large in the visual predictive check (Figure 2). However, this is not an uncommon phenomenon in studies with relatively small sample sizes.^38^ High relative standard errors on certain structural parameters and relatively high shrinkage values might also have occurred on account of a relatively small sample size and the sampling design. Caution is therefore warranted if the presented final model should be used for dose optimization. The secondary parameter estimates (Table 2) and the performed simulations (Figures 4 and 5) should also be interpreted with caution since a relatively high shrinkage might underestimate the variability of these parameters. However, the median values are not likely to be affected by shrinkage and should accurately show the important differences during recovery from the disease in pregnant women.

### Conclusions

The population pharmacokinetic properties of quinine in this study were described best by first-order absorption with two distribution compartments. Malaria had a significant effect on the pharmacokinetics of quinine. Quinine exposure was proportional to parasite density and increased by 50.7% in a typical patient with a total parasite biomass of 10^11^ infected erythrocytes compared with 10^7^ infected erythrocytes, and the quinine exposure during the first 8 h of treatment was 15% higher in patients with a body temperature on admission of 39°C compared with 36°C. Pregnancy-related covariates such as EGA or trimester did not significantly affect the pharmacokinetics of quinine. However, a non-significant trend of increased elimination clearance with trimester was observed. Quinine exposures in this study were approximately half those previously reported for non-pregnant patients in the literature. Plasma protein-binding studies are now needed to determine whether doses need to be increased in later pregnancy, particularly in women with low levels of parasitaemia who are afebrile.

## Funding

This study was an initiative of ‘Aid for poverty related diseases in developing countries’ and was co-financed by Médecins Sans Frontières and the European Commission. This investigation was part of the Wellcome Trust-Mahidol University-Oxford Tropical Medicine Research Programme, and the PKPDia collaboration, both supported by the Wellcome Trust of Great Britain. The drug assays were supported by the Malaria in Pregnancy (MIP) consortium, which is funded through a grant from the Bill and Melinda Gates Foundation to the Liverpool School of Tropical Medicine.

## Transparency declarations

None to declare.

The Wellcome Trust is a UK-based medical research charity and is independent of all drug companies. It has no financial links with the manufacturers of either the diagnostic tests or the drugs used in this study.
